# Individual and contextual factors associated with verbal bullying among Brazilian adolescents

**DOI:** 10.1186/s12887-015-0367-y

**Published:** 2015-05-01

**Authors:** Catarina Machado Azeredo, Renata Bertazzi Levy, Ricardo Araya, Paulo Rossi Menezes

**Affiliations:** Federal University of Uberlandia – School of Medicine (Universidade Federal de Uberlândia – Faculdade de Medicina), Av. Pará, n° 1720, Bloco 2U, sala 20. Campus Umuarama. Bairro Umuarama, Uberlândia, Minas Gerais 38.405-320 Brasil; University of Sao Paulo – School of Medicine (Universidade de São Paulo - Faculdade de Medicina), Av. Dr. Arnaldo 455, 1° andar, São Paulo, São Paulo 01246-903 Brasil; Department of Population Health, London School of Hygiene and Tropical Medicine, Centre for Global Mental Health, Keppel Street, London, WC1E 7HT UK

**Keywords:** Bullying, School, Adolescent, Associated factor, Multilevel modelling

## Abstract

**Background:**

Few studies have been carried out in low- middle-income countries assessing contextual characteristics associated with bullying. This study aimed to assess the relative importance of contextual (school and city) and individual-level factors to explain the variance in verbal bullying among a nationally representative sample of Brazilian adolescents.

**Methods:**

59,348 students from 1,453 schools and 26 state capitals and the Federal District participated in the National Survey of School Health among 9th Grade Students (PeNSE, 2009). We performed multilevel logistic regression in a three level model (individual, school and city).

**Results:**

The 30-day prevalence of verbal bullying among these students was 14.2%. We found that 1.8% and 0.3% of the total variance in bullying occurred at school-level and city-level, respectively, and 97.9% at individual-level. At city-level, all factors included failed to demonstrate a significant association with bullying (p <0.05) whereas at school-level, private schools presented more bullying than public schools (OR = 1.17, CI 1.04-1.31). At individual-level, male gender, younger age, not living with both parents, exposed to domestic violence, under or overweight were all associated with bullying.

**Conclusions:**

All socioeconomic indicators assessed contributed little to explain the variance in bullying at individual, school or city-level. Population subgroups at risk identified according to their individual profile could be targeted in future interventions in Brazil.

## Background

Bullying is a common form of aggression among adolescents worldwide. School-based surveys in high-income countries (HIC) have found average prevalence rates among adolescents ranging from 2% among girls aged 13 years in Italy to 32% within boys aged 11 years in Lithuania [1]. Surveys from low- and middle-income countries (LMIC) also show high prevalence, ranging from 7.8% in Tajikistan to 60.9% in Zambia [2]. In Latin America, relatively high prevalence has been observed in Chile (46.6%) and Venezuela (32.8%) [2]. A recent study from Brazil stratified bullying by frequency, with a prevalence of 5.4% reporting “often and very often being victimized”, and 25.4% “sometimes or rarely being victimized” in the last 30 days [3]. The wide variation in prevalence rates across countries is possibly related to the use of different methodologies and/or cultural or social factors [4,5].

Bullying behavior is of concern to both education and health systems, because of its deleterious consequences to victims. Bullying victims have higher risks of psychosocial problems, like low self-esteem, anxiety, depression [6] and suicide [7]; Moreover, victims present poorer school performance, and higher truancy and dropout rates [8,9].

There has been research suggesting that school bullying tends to cluster in classes, schools, cities and countries [10-15]. However, most research conducted so far shows that most of the variance is explained at an individual rather than higher level of aggregation. Notwithstanding this, risk factors should be studied in a comprehensive manner with adequate control of possible confounders [16] in order to get a broader understanding of the factors associated with bullying.

At the individual-level the following variables have been associated with bullying: male gender [17], younger age or earlier grades [18], being overweight or obese [19,20], exposure to physical abuse by adults [21-23], being from families that lack affection and discipline [24], and living only with one parent [25]. The role of ethnicity and individual socioeconomic status remains unclear [10,25-27].

The association of macroeconomic and social indicators with bullying has also been explored at a higher level (school, city and country). For instance it is argued that income inequality, is responsible for reducing social cohesion across communities and this is considered a preventive factor to reduce violence in general [28,29] including bullying [29]. It is also argued that socio-economic inequalities may contribute to increase abuse inflicted on those with less power [30].

At school-level, studies in HIC have found that poverty [30-33] or high socioeconomic disparities in schools [10] had a positive association with bullying. At country-level evidence suggests that income inequality, measured by the GINI index, a commonly used indicator of income inequality, also had a positive association with bullying in HIC [10,34]. Only two studies assessing concomitantly both individual- and contextual-level factors in LMIC have been reported so far [15,35]. However, none of them found an association between income or land ownership inequality or per capita income and bullying.

Violence is another characteristic that has been linked to bullying. The connection between violence and bullying in cities may be explained based on the social learning perspective that postulates that aggression is a behavior that may be learned from the environment in which individuals live. In other words, those who are exposed to violence in their communities learn that aggression, including bullying, may be an acceptable way to achieve goals, consequently developing normative beliefs, which may condone or legitimize aggression [15,36]. A study in Colombia showed that at school-level bullying was associated with higher levels of community violence and stronger beliefs in support of aggression. At city-level, those areas that had experienced more violence from the decades-old-armed conflict ^a^ had increased prevalence of bullying in schools, among 5th grade students [15]. On the other hand an international study in 15 LMIC [35] found no association between homicide rate at country-level and bullying. As the association between social and macroeconomic indicators and bullying has shown disparate findings in these studies in LMIC, more research seems warranted.

Bullying may take different forms, such as physical, verbal, relational/social or electronic (cyberbullying) [37,38]. In Brazil, studies have shown that verbal bullying is the most prevalent form of bullying [39,40], which is similar to the US and UK findings [41,42].

The associations previously described came from studies that analyzed factors associated with bullying as a whole, but may also be important to explore specific predictors of each form of bullying. At the individual-level, some gender differences were found, for example in associations with verbal and physical bullying [17]. Thus, it is possible that school and city-level predictors also vary according to the form of bullying assessed.

Brazil has the largest population in Latin America: 198.7 million people [43], with about 34.5 million aged 10–19 years and high school attendance, around 84% [44]. Brazil is classified as an upper-middle income country [43] and in spite of not being considered a poor country it has a high rate of income inequality (GINI index: 0.54) with substantial differences between geographical regions. Also, violence is an important cause of morbidity and mortality in Brazil, with most violent deaths being due to homicide and with an unequal distribution between geographic regions [45]. Social and income inequalities as well as violence are exceedingly common in Brazil and may be associated with bullying. Nonetheless, few studies on the prevalence and risk factors associated with bullying have been conducted in Brazil and all of them explored only individual-level factors [3,39,46,47].

Using a dataset from a Brazilian surveillance, which assessed verbal bullying among adolescents, we hypothesize that: schools and cities may contribute to explain some of the variance in verbal bullying among young people in Brazil. We also intend to explore associations between verbal bullying and individual characteristics, considering that previous research have shown that individual features are more strongly correlated with bullying.

## Methods

### Study population, sampling and data collection

The present study is based on data supplied by the National Survey of School Health among 9th Grade Students - PeNSE, which was carried out between March and July of 2009 [48].The main objective of this survey was to assess the risk and protective factors for health in adolescents from public and private schools in Brazil. The study population comprised adolescents attending the last year of private and public middle schools (grade 9) from 26 state capitals and the Federal District, which is one of the 27 federative units, in Brazil. In this paper, the Federal district will be analysed as a cluster in the same level as the other 26 state capitals.

The expected age range in 9th grade in Brazil is between 14 and 15 years old [49], but the real range is wider than this, especially due to students repeating grades or going to school before the age of 6.

The sample was selected from the 2007 School Census database using a complex design that included stratification per cluster and two-stage selection. The sampling strata considered each of the 26 state capitals and the Federal District. The primary sampling units (PSUs) were schools, and the secondary sampling units (SSUs) were school classes. The odds for school selection were proportional to the school size (total number of ninth-year classes), while the classes in each school were chosen by simple random selection. Two classes were selected from the schools with three or more ninth-year classes, and one class was selected from the schools with one or two ninth-year classes. All of the students enrolled in the selected classes were invited to participate in the study [48].

PeNSE 2009 used a self-reported structured questionnaire available in a Personal Digital Assistant^b^ device, which included the following thematic modules: socio-demographic characteristics, diet, body image, physical activity, smoking, use of alcohol and other drugs, support network (family and friends), sexual behavior, situations at home and school (including bullying), violence and accidents, and anthropometric measures (weight and height). The questionnaire was based on the Global School-based Student Health Survey/World Health Organization-GSHS/WHO and Youth Risk Behavior Surveillance System/Centers for Disease Control and Prevention- YRBSS/CDC.

From the total number of students selected for the sample (n = 63,411), 501 refused to participate, 1,937 did not report their gender and 1,625 did not answer the bullying question and thus were excluded from analysis (response rate 93.6%). The sample was re-weighted (based on gender losses and refusals) to represent students enrolled in the ninth year of schooling who attended regularly. Therefore, the analysis uses data corresponding to 59,348 students from 1,453 schools and 26 cities and the Federal District. Further details on the sampling procedures are available in the PeNSE report [48].

### Assessment of verbal bullying

Verbal victimization was measured using the question “In the past 30 days, how often have you been mocked, teased, called names or intimidated by one of your schoolmates so much that you were hurt/annoyed/upset/offended/ashamed?” Response options were “not at all”, “rarely”, “sometimes”, “most of the time”, and “always”. Students were categorized as victims of bullying if they reported being verbally bullied “sometimes” or more frequently. We assumed that the answer “sometimes” implies more than once, which is equivalent to the frequency of “two or more times within the last month”, a threshold recommended in previous literature [27,50].

### Description of independent variables

#### Individual-level measures

The following socio-demographic variables were considered in the analysis: gender; age range (≤13, 14, 15, ≥16 years); ethnicity/skin colour (white, black, brown or mixed-race, Asian, native Brazilian Indian); mother’s educational level (incomplete middle school, complete middle school, complete high-school, complete higher education). The socioeconomic-status was assessed as tertiles of the total scores of the Goods and Service index (GS score) [51]. The GS score was built based on the self-report of having: television, refrigerator, stove, microwave, washing machine, landline, mobile phone, DVD player, computer, car, bathroom inside the house and housemaid services. Each item was weighted by the inverse of the frequency of possession or presence in the total study sample. In addition, an imputation procedure was performed to attribute numerical values to missing score items as described previously [51]. The GS score of each student was obtained by adding the weighted scores.

The assessment of weight and height was performed by trained researchers and is described elsewhere [52]. After calculating Body Mass Index (BMI), we used the BMI index for age z-scores as an assessment of nutritional status. The nutritional status categories were: normal weight (≥ −2 z-score ≤ +1), thinness (z-score < −2), overweight (+1 > z-score ≤ +2) and obese (z-score > + 2) according to age and sex following recommendations by the World Health Organization [53].

Living arrangements were also evaluated and categorized into three groups: living with two parents, living with one parent (mother or father) or other arrangements. There was no differentiation between biological or adoptive parents. Domestic violence against the adolescent was evaluated from the question “in the past 30 days, how many times were you physically assaulted by an adult in your family”, categorized as “none”, “once”, or “two or more times”.

#### School-level contextual measures

Schools were classified as either public or private. We estimated a school’s socioeconomic level as the mean GS score of the children supplying data from each school. The GS score was distributed into tertiles. We used the coefficient of variation of mean GS score as a measure of a school’s socioeconomic disparity (i.e. a high value indicated a larger variation among students at the school). The coefficient of variation was also divided into tertiles.

#### Contextual city- level measures

The 2010 per capita income of each city obtained from the Brazilian Institute of Geography and Statistics – IBGE (http://www.ibge.gov.br/home/estatistica/populacao/censo2010/indicadores_sociais_municipais/tabelas_pdf/tab16.pdf), divided in tertiles. It was used as an indicator of overall city income. In addition we obtained GINI indices for each capital, also from IBGE system (2010=http://tabnet.datasus.gov.br/cgi/idb2011/b09capc.htm), which were subsequently divided into tertiles. A GINI index of zero represents perfect income equality and a score of 1 maximum inequality. Violence in the capital was assessed by homicide rate (2009) obtained from the Brazilian Department of Public Health Information [54] and it was also divided into tertiles.

### Statistical analyses

First the prevalence and distribution of the covariates of interest were analysed incorporating the complex sample design of PeNSE 2009. The relationships between being a victim of verbal bullying and individual-level, school-level and city-level characteristics were assessed by three-level logistic regression models, whereby crude and adjusted odds ratios (ORs) with the corresponding 95% confidence intervals were obtained. The cut-off point for statistical significance was arbitrarily established as p < 0.05.

We performed multilevel analysis at different levels taking into consideration the data hierarchy (students nested in schools and schools nested in cities) to analyze the proportion of the variance explained at different levels using regression models [55].

Initially we calculated the crude variance coefficients representing between-city and between-school variance in exposure to bullying victimization without considering any individual, school, or city variables (empty model). This model indicates the crude amount of clustering of bullying by school and city. We used the ‘latent variable method’ to obtain intraclass correlation coefficients (ICC), assuming that an individual’s risk of bullying follows a logistic distribution, with individual-level variance equals to 3.29 [56,57].

In a second step, we analyzed the association between each covariate with bullying, without additional adjustments for other variables (Unadjusted model). Then, the influence of all the individual-level covariates on the outcome was examined (model 1). In Model 2 we simultaneously examined the influence of all covariates, student, school and city-level. We also verified the proportional change in variance of bullying across schools and cities for models 1 and 2 [58].

All the analyses were performed using Stata SE version 13 [59].

### Ethical aspects

PeNSE 2009 was approved by the National Commission of Research Ethics (ComissãoNacional de ÉticaemPesquisa – Conep), record no. 11.537. It was performed in accordance with the Declaration of Helsinki and all participants gave their informed consent. Database was made publicly available on an IBGE website without any information that could identify subjects.

## Results

The descriptive analysis of individual, school and city-level characteristics are presented in Table 1. Students were predominantly within the age range between 14-15years-old (65.39%), white (40.23%) or brown/mixed race (39.11%), and lived with both parents (58.06%). The Goods and Services (GS) Score ranged from 0 to 22, and the mean was 11.92 (SE = 0.07). More than two-tenths of students were overweight and obese; nearly 10% had suffered violence at least once from someone in the family within the last 30 days (Table 1). The prevalence of verbal bullying among students was 14.2% (95% CI: 13.60 -14.75). Most of schools were public (75.95%), the mean of GS score ranged from 5.70 to 21.14 among schools, and the coefficient of variance of GS score ranged from 9.14% to 64.17% among schools.

**Table 1:** **Description of the sample: Individual, School and City level characteristics in the National Survey of Schoolchildren’s Health (PENSE 2009) dataset**

**Individual variables (n=59,348)**	**% (95% CI)**
**Gender**	
Female	52.76 (52.09 - 53.43)
Male	47.24 (46.57- 47.91)
**Age band**	
<=13y	24.53 (23.46 - 25.63)
14 y	47.19 (46.02- 48.36)
15y	18.20 (17.16 - 19.29)
>=16y	10.08 (9.33- 10.88)
**Race/color**	
White	40.23 (38.94 -41.53)
Brown or mixed-race	39.11 (38.00 - 40.24)
Black	12.83 (12.20 - 13.48)
Asian	3.74 (3.45 - 4.06)
Native Brazilian indian	4.10 (3.82 - 4.37)
**Maternal Educational level**	
Incomplete middle school	31.70 (30.32 - 33.10)
Complete middle school	16.89 (16.27 - 17.53)
Complete high school	31.55 (30.33 - 32.80)
Complete higher education	19.86 (18.54 -21.26)
**Family Arrangement**	
Two parents	58.37 (57.42- 59.31)
Only one parent	36.38 (35.54 -37.24)
Other	5.25 (04.92- 05.59)
**Victim of domestic violence**	
None	90.49 (89.98- 90.98)
Once	5.17 (04.84 - 05.53)
Twice or more	4.33 (04.01 - 04.68)
**Nutritional Status**	
Underweight	2.87 (2.64 - 03.12)
Eutrophic	74.00 (73.26 - 74.73)
Overweight	15.93 (15.37 - 16.51)
Obese	7.20 (6.86 - 07.56)
Bullying victim	14.20 (13.60 -14.75 )
GS score (Mean (SE))	12.02 (0.067)
**School Characteristics (n=1,453)**	**Total % (95% C.I.)**
Public	75.95 (74.52 - 77.32)
Private	24.05 (22.68 - 25.48)
Tertiles of GS score (Mean (SE))	11.92 (0.07)
Coefficient of variation of GS score (Mean (SE))	28.3 (0.21)
**City Characteristics (n=27)**	**Total Mean (SE)**
Per capita Income	19,949.58 (15,157.06)
Homicide rate^a^	37.92 (3.03)
GINI Index	0.53 (0.01)

^a^rate per 100,000 inhabitants.

95% (CI) = 95% Confidence Intervals.

There was wide variation in the prevalence of bullying among schools, ranging from 1.8% to 40.0%, and between cities ranged from 10.9% (Cuiabá) to 16.3% (Curitiba). In the empty model, the ICC showed that 1.8% and 0.3% of the total variance in verbal bullying occurred at school-level and city-level, respectively, and 97.9% at individual-level. These variances were statistically significant, showing clustering of bullying at these levels. The inclusion of covariates at individual-level in model 1 were able to explain 33.3% of the school-level variance. The inclusion of covariates at school and city-level in model 2 were not able to explain the school and city-level variance (Table 2).

**Table 2:** **Relative contribution of City, School and Individual level factors in the variance of bullying across Brazilian cities (level 1: individuals n = 59348, level 2: school n = 1453, level: 3 city n = 27)**

**Model**	**Variance**	**S.E.**	**ICC, %**	**Proportional change in variance, %**
**Empty model**				
City level	0.01	0.004	0.3	
School level	0.06	0.010	1.8	
Individual level	3.29	---	97.9	
Total variance	3.36		100.0	
**Model 1 (Individual-level variables)**				
City level	0.01	0.005		0.0
School level	0.04	0.012		33.3
Individual level	3.29	---		
Total variance	3.34			
**Model 2 (full model with per capita income)**				
City level	0.01	0.004		0.0
School level	0.04	0.012		33.3
Individual level	3.29	---		
Total variance	3.34			

### Factors associated with verbal bullying victimization

#### Contextual-level characteristics (school- and city-level)

In the unadjusted model schools with higher mean of GS scores (third tertile) presented a higher likelihood of victimization and private schools were also more likely to report bullying. In the adjusted model, there were no associations between bullying and the school-level mean of GS scores or the coefficient of variation (representing socio-economic inequalities). However private schools remained associated with more bullying victimization in the fully adjusted model (see Table 3). At the city-level, per capita income was positively associated with bullying only in the unadjusted model. Neither income inequality (GINI index), nor the homicide rate were associated with bullying.

**Table 3:** **Odds Ratios for association between individual-, school- and city-level variables with self-reported verbal bullying victimization - Multilevel Analysis**

**Variable**	**Bullying (%)**	**Unadjusted model**	**Model 1**	**Model 2**
		**Crude OR (95% CI)**	**OR (95% CI)**	**OR (95% CI)**
**INDIVIDUAL-LEVEL**				
**Gender**				
Female	13.1	1	1	1
Male	15.4	**1.33 (1.26-1.39)**	**1.39 (1.32-1.47)**	**1.39 (1.31-1.47)**
**Age**				
16y or more	12.6	**1**	1	1
15y	12.3	**1.10 (1.00-1.21)**	**1.21 (1.08-1.36)**	**1.21 (1.07- 1.35)**
14y	14.7	**1.25 (1.15- 1.37)**	**1.50 (1.35-1.66)**	**1.49 (1.34 -1.66)**
≤13y	15.3	**1.34 (1.22 -1.47)**	**1.63 (1.46-1.82)**	**1.62 (1.45 -1.82)**
**Race/color**				
White	14.7	1	1	1
Brown or mixed-race	13.4	0.91 (0.86-0.97)	0.94 (0.88-1.00)	0.94 (0.89 -1.01)
Black	14.6	1.03 (0.95-1.12)	1.02 (0.93-1.12)	1.03 (0.94 -1.13)
Asian	14.9	0.98 (0.86-1.11)	1.05 (0.91-1.21)	1.05 (0.91 -1.21)
Native Brazilian Indian	16.2	**1.14 (1.02-1.28)**	**1.17 (1.03-1.34)**	**1.17 (1.03 -1.34)**
**Maternal Educational level**				
Complete higher education	14.5	1	1	1
Complete high school	14.2	1.00 (0.92-1.07)	1.01 (0.94-1.10)	1.03 (0.94 -1.12)
Complete middle school	14.1	0.99 (0.90-1.08)	1.03 (0.94-1.14)	1.05 (0.95- 1.16)
Incomplete middle school	14.4	0.99 (0.91-1.07)	1.08 (0.99-1.18)	1.10 (1.00- 1.21)
**Tertiles GS score**				
1° tertile	13.6	1	1	1
2° tertile	14.8	1.03 (0.97-1.09)	0.99 (0.93-1.07)	0.98 (0.91- 1.05)
3° tertile	14.1	1.01 (0.95-1.07)	0.95 (0.87-1.02)	0.91 (0.84- 0.99)
**Family Arrangement**				
Live with both parents	13.3	1	1	1
Live with only one parent	15.4	**1.10 (1.04-1.15)**	**1.07(1.01-1.13)**	**1.07 (1.01-1.13)**
Other arrangements	14.2	**1.11 (1.01-1.23)**	**1.14 (1.01-1.28)**	**1.14 (1.02- 1.29)**
**Domestic violence**				
None	12.8	1	1	1
Once	22.5	**2.09 (1.91-2.28)**	**2.09 (1.89-2.31)**	**2.09 (1.89- 2.31)**
Twice or more	29.4	**3.35 (3.05-3.67)**	**3.41 (3.08-3.79)**	**3.37 (3.04- 3.74)**
**Nutritional status**				
Eutrophic	13.3	1	1	1
Underweight	16.0	**1.44 (1.26-1.64)**	**1.50 (1.29-1.74)**	**1.50 (1.29- 1.74)**
Overweight	15.4	**1.16 (1.09-1.24)**	**1.17 (1.09-1.26)**	**1.17 (1.08- 1.26)**
Obese	19.3	**1.45 (1.33-1.58)**	**1.41 (1.27-1.56)**	**1.40 (1.27- 1.55)**
**SCHOOL-LEVEL**				
**Administrative status**				
Public	14.0	1		1
Private	14.8	**1.11 (1.04-1.18)**		**1.17 (1.04-1.31)**
**GS score** ^**a**^ **(school mean)**				
1^st^tertile	13.5	1		1
2^**nd**^ tertile	14.7	1.08 (1.00-1.16)		1.02 (0.94-1.12)
3^**rd**^ tertile	14.4	**1.11 (1.04-1.18)**		0.96 (0.85- 1.08)
**Coefficient of variation**				
1^**st**^ tertile (GS score)	14.6	1		1
2^**nd**^ tertil	14.1	1.03 (0.96-1.11)		1.11 (1.02-1.21)
3^**rd**^ tertil	13.8	0.94 (0.88-1.02)		1.07 (0.97- 1.18)
**CITY-LEVEL**				
**Homicide rate**				
1^**st**^ tertile	14.9	1		1
2^**nd**^ tertile	14.1	0.91 (0.81-1.02)		0.94 (0.83-1.05)
3^**rd**^ tertile	13.4	0.99 (0.89-1.11)		1.00 (0.89-1.12)
**GINI Index**				
1^**st**^ tertile	14.3	1		1
2^**nd**^ tertile	13.2	0.99 (0.89-1.12)		0.94 (0.83- 1.06)
3^**rd**^ tertile	13.7	1.02 (0.91-1.15)		0.96 (0.85- 1.08)
**Tertiles of** ***per capita*** **income**				
1^**st**^ tertile	13.3	1		1
2^**nd**^ tertile	14.5	0.98 (0.88-1.09)		0.98 (0.87- 1.10)
3^**rd**^ tertile	15.9	**1.13 (1.02-1.26)**		1.11 (0.97- 1.27)

Model 1: adjusted by all individual-level covariates; Model 2: adjusted by individual-level, school-level and city-level covariates. ^a^Goods and Services Score.

OR = Odds Ratio.

Bold data reflect statistical significance (p<.05).

#### Individual-level characteristics

With regards to the association between verbal bullying and individual-level characteristics in adjusted models, there were significant associations for: male gender (OR = **1.39, IC 1.32-1.47)**, younger age (OR = **1.61, IC1.44-1.80)**, native ethnic groups (OR = **1.17, CI 1.03 -1.34),** not living with both parents (OR_one parent_ = **1.07, CI 1.01-1.13**; OR_other_ = **1.14, CI 1.02-1.29)**, victim of violence within the family at least once in the last month (OR _once_ = **2.09, IC 1.89-2.31**; OR _twice or more_ = **3.42, IC 3.08-3.79)**, overweight (OR = **1.17, IC 1.08-1.26)**, obesity (OR = **1.41, IC 1.27-1.56)** and underweight (OR = **1.50, IC 1.29-1.73)** (Table 3).

## Discussion

This is the first large multilevel study in Brazil to investigate the association between verbal bullying and individual and contextual covariates among a representative sample of Brazilian adolescents. The prevalence of self-reported verbal bullying was 14.2%, and most of the variance in bullying occurred at individual-level. Even though, the proportion of the variance explained at school level was fairly small, we found that students from private schools were more likely to report verbal bullying. At city level, all factors failed to demonstrate an association with verbal bullying. At the individual-level, male gender, young, not living with both parents, exposed to domestic violence, underweight, overweight or obese were significantly associated with bullying.

The relative contribution of school and city-level in verbal bullying variance among this large sample of Brazilian students was of small magnitude but in keeping with other studies ranging from 0.6 to 4.0% within schools, and from 1.7 to 9.1% within cities [12,14,15,30,60]. However it is noteworthy that we found a positive association between private schools and bullying. In Brazil there is a distinct socioeconomic difference between those who attend public and private schools, with the lower socioeconomic classes attending public schools. Thus, school status is often considered a socioeconomic indicator in Brazil. However, this association needs to be treated with caution because we did not find an association between socioeconomic level according to the individual GS score or maternal education and bullying. Further research is needed to explore what these complex variables actually represent. Chaux et al. [15] found similar association in Colombia, but stressed that it is not possible to state whether there is more awareness about bullying in private schools [15].

The lack of association between income inequality aggregated at school-level with verbal bullying, which had been previously reported to bullying in the literature [10], might be due to the fact that Brazilian schools are quite homogeneous in this respect.

Despite the fact that we have not found significant association between verbal bullying and income inequality at city-level, this association cannot be ruled out. A limitation of our study is that the indicator of inequality could not be measured at the most appropriate level. Most inequalities in Brazil are more noticeable when comparing large cities and small towns, urban and rural areas and within cities comparing central and peripheral areas but not when comparing state capitals. Nonetheless, we were unable to obtain this data because it was considered that this information could lead to the identification of the schools. It is also possible that the association between inequality and bullying may be more related to other forms of bullying or attributable to individual level factors, such as age and sex, obesity and being victim of domestic violence, rather than contextual variables. Regardless these possibilities, findings from other LMIC have not supported the association between inequality and bullying [15,35].

No association between either poverty or homicide rate and verbal bullying were found in Brazilian data. This suggests that regardless of the theoretical assumptions and findings linking violence and bullying [29] in Brazilian settings these features do not seem to explain verbal bullying variance among adolescents. The lack of association might be attributable to the measurement of verbal bullying. Perhaps if we had measured bullying as a summary of all its forms (physical, verbal, relational, cyberbullying) these associations could have been found. Despite this, our result is in keeping with those reported by Wilson et al., [35] in LMICs, for bullying measured as a whole.

Overall the associations between individual factors and verbal bullying were consistent with the literature, but cannot be extrapolated to other forms of bullying. Boys were more likely to report victimization, as reported by others especially for physical and verbal bullying, which are overt forms of aggression [17]. It is possible that girls may be more subtle in their bullying or less willing to report it than boys [31]. Young students reported victimization more often, something that may reflect the power imbalance when students of different ages attend the same classes. Those adolescents not living with both parents were more likely to report victimization. Growing up in single-parent families seems to be associated with more bullying victimization [61]. Jablonska and Lindberg [62] stated that social and economic characteristics and physical and mental problems of the parents are more commonly found in single parent households, perhaps explaining, at least in part, the association between single parents and bullying. However, our data do not allow us to examine these causal pathways.

Native Brazilian indian students were more likely to report victimization than white students, and this was the only race/color variable associated with verbal bullying in our sample. Being bullied is often related to being different, thus native individuals, representing a minority group in the Brazilian population (and in the sample of students, 4.1%), may be perceived as different for a number of reasons, including different cultural and social norms [63].

Individuals who reported violence at home were much more likely to report verbal bullying victimization. Although we cannot establish the causal direction of this association, it has been reported that domestic violence increases anxiety and depressive symptoms, low self-esteem and social isolation, which might interfere with the social skills of these adolescents who could become easy targets for bullies [64,65].

Underweight, overweight and obese adolescents reported more bullying than their eutrophic counterparts, something that had been previously reported in the literature [20,66]. This study, however, uses a more accurate measure of weight and height that were directly assessed and not self-reported by students, as in previous [19,20,67]. It has been reported that those adolescents whose body weight does not fit with what is seen as desirable in a particular setting (ideal body shape) have greater difficulty socializing in school and poorer psychological well-being, something that increases their likelihood to be bullied [68].

This study has some limitations. First, the measure of bullying based on a single-item is rather limiting but other studies have also had a similar problem. This question seeks to capture those individuals who had experienced negative feelings as a result of bullying. Students classified as victims in this study might be more severe cases of bullying or those who were unable to cope well with the situation. Therefore it is possible that the prevalence of verbal bullying as measure in this study may have been underestimated. Additionally, the associations found or not found, can only be stated for verbal bullying and might differ for other forms of bullying or for bullying measured as a summary of its different forms. However, the lack of these measures in the surveillance used prevented us from exploring other associations.

The second limitation is the use of aggregated measures to assess school characteristics instead of using proper contextual level measures [69]. Unfortunately contextual measures were not available at school level. However, some authors have argued that it is valid to use summarized or aggregated measures to assess contextual characteristics in multilevel analyses [70]. Third, it was not possible to include probability weights in the multilevel regression analysis using Stata, since the full model could not achieve convergence using Gllamm commands. Nevertheless, the sample structure was considered in the analyses using XT commands in Stata, and comparing three-levels models with and without sample weights the results were similar. Finally, when interpreting the findings of this study, the cross-sectional nature of our data did not allow us to make causal inferences.

The major strength of this study is the large random sample, representative of Brazilian schoolchildren of 9^th^ grade and the good response rate. The direct measurement of height and weight of students rather than self-reported measures also reduced potential measurement bias [71].

## Conclusion

This study provided evidence that verbal bullying variance at school and city-level was small, and most of the variance was accounted for at the individual level. This does not mean school or city-level lacks importance, but may suggest that city boundaries comparing only state capitals do not capture differences that shape the relevant environment for bullying occurrence [56]. Those contextual variables analyzed contributed little to explain the variance in verbal bullying, between schools and cities, among 9th grade school students in Brazil. However, the lack of association between inequality at city-level and verbal bullying should be read with caution, since measurement concerns were described.

Those individual variables strongly associated with verbal bullying might allow the identification of possible higher risk sub-groups that could be targeted for interventions in the future and denote the role of family arrangements and violence in verbal bullying victimization. The profile of vulnerable adolescents to verbal bullying in Brazil can guide policy makers, teachers and school staff on bullying prevention.

Finally studies exploring associations between other forms of bullying and other contextual variables, measured at rural/urban or central/peripheral neighborhoods; or comparing state capitals with small towns; including other family and school characteristics, which are more proximal to individuals, may provide a more accurate picture of the possible influence of contextual-level variables on bullying in Brazil.

## End notes

^a^Decades-old-armed conflict has happened among left-wing guerrilla groups, right-wing para-militaries and the national-armed forces in Colombia.

^b^available at: http://www.ibge.gov.br/home/estatistica/populacao/pense_avaliacao_nutricional_2009/questionario.pdf.

## Abbreviations

HIC: High-income countries
LMIC: Low- and middle-income countries
PeNSE: National Survey of Health among 9th Grade Students
PSUs: Primary sampling units
SSUs: Secondary sampling units
GS score: Goods and Service scores
OR: Odds ratio
ICC: Intraclass correlation coefficients
